# Is It Safe to Perform Lung Surgery During the Coronavirus Pandemic?

**DOI:** 10.7759/cureus.9749

**Published:** 2020-08-14

**Authors:** Anupam K Gupta, Max Jackson, Thomas Genuit, John Roberts

**Affiliations:** 1 Minimally Invasive Surgery, University of Miami Hospital, Miami, USA; 2 Thoracic Surgery, Bethesda Hospital East, Charles E. Schmidt College of Medicine, Boynton Beach, USA; 3 Thoracic Surgery, Delray Medical Center, Johns Hopkins University, Delray Beach, USA

**Keywords:** covid-19, lung surgery, thoracic surgery, lung cancer

## Abstract

Background: Coronavirus disease (COVID-19) patients are rapidly growing in our community. Patients with compromised lungs and older age are supposedly at high risk of poor outcomes with COVID-19. We aimed to evaluate the COVID-19 impact on lung surgery during this pandemic at our hospital.

Methodology: This is a retrospective study of all lung surgery patients at our hospital in Boca Raton over three months (February to April 2020). All patients who remained for at least one-day inpatient post-lung surgery were assessed to see if they had an increased incidence of coronavirus infection during the hospital stay or at the follow-up office visit.

Results: A total of 44 patients underwent thoracic surgery. It was found that there was no incidence of coronavirus infection in these patients.

Conclusion: With adequate precautions, older patients can undergo lung surgery during this pandemic. There was no incidence of COVID-19 found among the patients during the hospital stay or at the first follow-up in the office. Also, the postoperative course was not adversely affected.

## Introduction

Coronavirus disease 2019 (COVID-19) was identified in Wuhan, China, in December of 2019 and has subsequently grown into a global pandemic [1-5]. As of April 20, 2020, the world had over 2,463,357 reported cases. The United States of America had over 750,000 cases, with the state of Florida having 26,660 cases [5-10]. Palm Beach County had over 2,110 cases, with 121 deaths [5,10]. As of April 20, 2020, we have reported 55 cases requiring intensive care unit (ICU) care at our tertiary medical center Boca Raton Regional Hospital (400 bedded hospital) in Florida and over 250 coronavirus positive patients. There is no clear evidence as to why some people are more susceptible to the coronavirus infection [8,9]. This study was conducted to evaluate whether it is safe to perform lung surgery in the community where there is a rapidly increasing number of coronavirus patients.

## Materials and methods

Ours is a retrospective study performed at Boca Raton Regional Hospital, Palm Beach County, in Florida. Boca Raton Regional Hospital is a 400 bedded tertiary level center. From February 1, 2020, to April 14, 2020, the patients who underwent lung surgery were evaluated for the incidence of coronavirus infection during the hospital stay and on postoperative follow-up. Patients underwent surgery after triage and if they fulfilled guidelines set by the American College of Surgeons and hospital review board, so that hospital resources were not exhausted during an increasing number of COVID cases in our community.

The patients underwent screening for coronavirus infection based on history and imaging (chest x-ray and computed tomography), and a suspected person underwent nasal polymerase chain reaction (PCR) for coronavirus. A single positive test was to categorize the patient as a coronavirus-infected patient; in patients with high clinical suspicion, two consecutive negative PCR test results ruled out coronavirus infection.

We evaluated electronic medical records for demographics, type of surgery, laboratory, radiological data, symptoms, and duration of stay in the hospital. The data collected and presented as absolute numbers and percentages. Use of personal protective equipment with regular handwashing and clinical care were followed as per guidelines set by the Society of Thoracic Surgeons during the care of these patients.

The inclusion criterion for selecting the patients was as follows: patients undergoing lung/thoracic surgery needing at least one-day hospitalization from February 1, 2020, to April 14, 2020. The exclusion criteria were as follows: (1) non-thoracic surgery patients, (2) patients who did not have any surgery performed, and (3) patients who underwent procedures on an outpatient basis.

## Results

During the duration of the study, we had two resident doctors and four nurses from the thoracic surgery team who tested positive for coronavirus infection on the nasal PCR test. Nine healthcare providers involved in patient care underwent quarantine for two weeks after the development of symptoms/positive PCR tests during the duration of this study.

A total of 44 patients underwent lung/thoracic surgery from February 1 to April 14, 2020 (Table 1). A total of 27 lung surgeries (wedge/lobectomy/pneumonectomy) were for non-small cell cancer of the lung. Six underwent video-assisted thoracic surgery for empyema. The mean age of patients in the study was 72.3 years. Thirty-nine patients were older than 65 years. Patients’ postoperative hospital stay ranged from 1 to 32 days (prolonged stay for one patient was due to complications secondary to a leak post-esophageal surgery; however, the patient was negative for COVID-19 on the nasal PCR test), with a mean of 7.68 days. The mean ICU stay duration was 1.70 days. During the hospital stay, each of the patients received chest radiographs/computed tomography as needed for their management.

**Table 1:** Demographics and length of hospital stay VATS, video-assisted thoracoscopic surgery; ICU, intensive care unit; LOS, length of stay; EBUS, endobronchial ultrasound. *Diaphragm plication, Clagett revision, VATS, pneumonectomy, VATS parietal pleurectomy, thoracotomy with trachea resection.

Case	N	Age (years, mean ± SD)	ICU LOS (days, mean ± SD)	Hospital LOS (days, mean ± SD)
EBUS/bronchoscopy/mediastinoscopy	6	75 ± 3.52	0.83 ± 2.04	4.83 ± 5.56
Thoracotomy, lobectomy	3	68 ± 2.65	3.67 ± 3.51	9.33 ± 2.52
VATS lobectomy	12	68.92 ± 6.67	1.42 ± 4.91	8.58 ± 10.20
VATS wedge resection	11	79.64 ± 5.71	1.64 ± 3.32	6.64 ± 2.87
VATS decortication	4	76.50 ± 9.04	3.25 ± 6.50	9.50 ± 5.07
VATS pleurodesis	2	55	0.00	5.50 ± 2.12
Ivor-Lewis esophagectomy	1	72	3	28
Other*	5	70.00 ± 4.90	1.60 ± 2.30	5.60 ± 2.70
All cases	44	72.61 ± 8.00	1.70 ± 3.75	7.68 ± 6.89

All patients’ follow-up was within two weeks from the time of discharge. There was a 100% follow-up at the office and the patients underwent evaluation for symptoms, imaging, and blood work as needed. We had no patients who needed further assessment or testing for COVID-19 on follow-up. One patient needed readmission for breathlessness secondary to pneumothorax; five patients developed symptoms/radiographic signs of COVID-19 and tested negative for COVID-19 with two consecutive nasal PCR tests. There were zero incidences of COVID-19-positive cases, and the mean duration of hospital stay remained similar when compared to prior months.

## Discussion

In January 2020, the first case of COVID-19 was reported in the United States [1]. Subsequently, there has been a rapid increase in cases in the United States and our community in Florida [1,2]. The mode of transmission is primarily respiratory droplets and close contact [3-9]. Coronavirus binds to angiotensin-converting enzyme receptor-2 and dipeptidyl peptidase-4 receptors on bronchial epithelial cells and Type 2 pneumocytes [6-9]. Symptoms can vary from being asymptomatic to having a dry cough, breathlessness, and even death [6-9]. The current Centers for Disease Control (CDC) report suggests that all demographics of the global population can get infected with coronavirus [9-12]. The CDC classifies individuals over more than 65 years of age who are at risk of more severe disease than younger people [9-12]. There is also a report that patients with underlying chronic pulmonary disease and lung cancer are at an increased risk of severe infection [9-12]. Diagnosis is confirmed by real-time reverse transcriptase PCR from respiratory and blood samples [8-10]. Imaging like chest x-ray showing reticular shadow and small patchy opacities, and computed tomography findings of bilateral multilobar ground-glass opacities are suggestive of coronavirus infection [8-10]. Blood work usually shows a normal or decreased leukocyte count [5-10]. Currently, there is no effective treatment for coronavirus infection, and because of the strain on hospital resources to manage the COVID-19 pandemic, hospitals have reduced elective cases [9,10,12]. While some patients can delay lung surgery, other patients’ failure to receive surgical care can have a severe impact on their quality of life [10,11]. With thoracic oncology cases, the patients are at a high risk because of their impaired lung function and can have potentially poor outcomes [10,11].

A set of guidelines by the Thoracic Surgery Outcomes Research Network to triage patients is being followed at most centers around America; however, these guidelines are based on assumptions [10,11].

## Conclusions

In older patients with lung cancer, lung surgery if carried out with adequate precautions does not lead to an increased incidence of COVID-19, as noticed in the patients at our hospital. Also, the postoperative course is not adversely affected in a community with a rising number of COVID-19 patients, as there was no incidence of COVID-19 in the hospital or on the follow-up visit in our older patients with compromised lungs. One limitation of this study was that our patient population is small, and further studies would help in establishing the safety of lung surgery during this pandemic.
